# Biodegradable and Mechanically Resilient Recombinant Collagen/PEG/Catechol Cryogel Hemostat for Deep Non-Compressible Hemorrhage and Wound Healing

**DOI:** 10.3390/gels11060445

**Published:** 2025-06-10

**Authors:** Yuanzhe Zhang, Tianyu Yao, Ru Xu, Pei Ma, Jing Zhao, Yu Mi

**Affiliations:** 1 Engineering Research Center of Western Resource Innovation Medicine Green Manufacturing, Ministry of Education, School of Chemical Engineering, Northwest University, Xi’an 710127, China; 202221124@stumail.nwu.edu.cn (Y.Z.); t.yao@nwu.edu.cn (T.Y.); mapei@nwu.edu.cn (P.M.); 2 Shaanxi Key Laboratory of Biomaterials and Synthetic Biology, Shaanxi R&D Center of Biomaterials and Fermentation Engineering, School of Chemical Engineering, Northwest University, Xi’an 710127, China; 3 Biotechnology & Biomedicine Research Institute, Northwest University, Xi’an 710127, China; 4 Xi’an Giant Biogene Technology Co., Ltd., Xi’an 710069, China; xuru560@163.com

**Keywords:** recombinant collagen CF-1552, cryogel, hemostasis, shape memory recovery, wound healing

## Abstract

Traumatic non-compressible hemorrhage and subsequent wound management remain critical challenges in military and civilian settings to this day. Cryogels have emerged as promising hemostatic materials for non-compressible hemorrhage due to their blood-triggered shape recovery. In this study, a biodegradable and mechanically resilient cryogel (CF/PD) was produced via cryopolymerization, employing methacrylated recombinant collagen as a macromolecular crosslinker alongside poly (ethylene glycol) diacrylate (PEGDA) and dopamine methacrylate (DMA). With its interpenetrating macro-porous structure and high hydrophilicity, the CF/PD rapidly absorbs blood and returns to its original shape within 1.5 s. In a rat liver defect model, CF/PD outperformed commercially available gelatin sponges, reducing hemostasis time by 74.4% and blood loss by 76.5%. Moreover, CF/PD cryogels facilitate in situ tissue regeneration by virtue of the bioactivity and degradability of recombinant collagen. This work establishes a bioactive recombinant collagen-driven cryogel platform, offering a transformative solution for managing non-compressible hemorrhage while enabling tissue regeneration.

## 1. Introduction

Uncontrolled bleeding causes approximately 80% of deaths on the battlefield [1,2]. Except in military conflicts, deaths due to severe bleeding account for 40% of trauma-related fatalities in hospital settings [3]. About 30–40% of these traumatic deaths occur within 24 h of trauma [1,4]. Therefore, rapid hemostasis is critical to effectively save a patient’s life when injury occurs. Current hemostatic agents, such as various hemostatic dressings [5,6] and hemostatic bandages [7,8,9], are effective in controlling bleeding at limbs and superficial wounds; however, these materials have significant limitations in managing deep, non-compressible hemorrhages, especially those occurring at vital organs and body junctions. Cryogels have interconnected macro-porous networks, high hydrophilicity, and shape memory properties, making them suitable for the hemostasis of deep non-compressible wounds [10,11,12,13,14]. The cryogels can be compressed and inserted into wounds, and after absorbing blood, they can quickly recover to their original shape and mechanically seal the wound.

However, cryogels prepared using natural polymers such as chitosan, alginate, and dextrin are usually crosslinked by non-covalent bonds such as hydrogen bond, Schiff base bond, and chain entanglement, which were shown to have limited mechanical strength that cannot exert sufficient physical pressure to stop bleeding [15,16]. In order to provide sufficient mechanical resilience to quickly seal the wound, nanoparticles such as polydopamine and bioactive glass were introduced to the cryogel systems [15,17]. In previous studies, we developed polydopamine nanoparticle (CHOP) and bioactive glass-loaded chitosan/oxidized dextran/recombinant collagen (AOM) cryogels for hemostasis of non-compressible wounds [15,17]. The incorporated nanoparticles significantly increased the mechanical strength and reduced the shape recovery time; however, the nanoparticles raise concerns about nanotoxicity, which is still under extensive study, and the metabolism and elimination profile of many nanoparticles have not been fully elucidated.

Another strategy to improve the mechanical resilience of cryogels for rapid shape recovery is forming covalent crosslinking and interpenetrating polymer networks (IPNs) through cryopolymerization. Cryogels produced by this method were shown to have high mechanical strength that can withstand multiple compressions and effectively seal the wound [18]. In addition, for cryogels prepared by cryopolymerization, the gelation occurs in the interstices of ice crystal growth, allowing extended ice crystal growth, thereby producing cryogels with larger, more interconnected porous structures that allow more rapid absorption of blood and faster shape recovery [19,20]. However, most cryogels produced by the cryopolymerization method have poor degradability [21,22,23].

Collagen is widely used in hemostasis due to its biocompatibility, biodegradability, and low immunogenicity [24,25,26,27,28,29,30,31]. It actively promotes blood clot formation by activating platelets and initiating the coagulation cascade [24,26]. The degradation products of collagen provide a nourishing microenvironment to support tissue repair [32]. Recombinant collagen could avoid the risk of immunogenicity and viral hazards of animal-derived collagen while offering better water solubility to facilitate fast gelation and high uniformity. By modifying recombinant collagen with double bonds, it can function as a biodegradable macromolecular crosslinker for the hemostatic material prepared by cryopolymerization.

Hemostatic agents containing catechol moieties have wet tissue adhesion wound sites, which prevent them from slipping out [33]. Also, catechol groups promote coagulation by aggregating platelets [34]. Additionally, catechol-functionalized biomaterials exhibit inherent biocompatibility and broad-spectrum antimicrobial activity [35]. Poly (ethylene glycol) (PEG) is a biocompatible material that has low immunogenicity and has been approved by the U.S. FDA as a GlycoPEGylated material (N8-GP). As a highly hydrophilic polymer, PEG was shown to improve the water solubility and mechanical strength of PEG-based material and promote hemostasis [36]. In a previous study by Huang et al., gelatin/polydopamine (PDA) cryogels were shown to exhibit a 10-fold longer shape recovery time in blood compared to Phosphate Buffer Saline (PBS) [18], suggesting a trade-off between cell adhesion and blood absorption speed. The incorporation of PEG into the collagen-based cryogel may overcome this drawback and reduce the shape recovery time, thereby improving hemostatic efficiency in penetrating wounds.

**Scheme 1:**
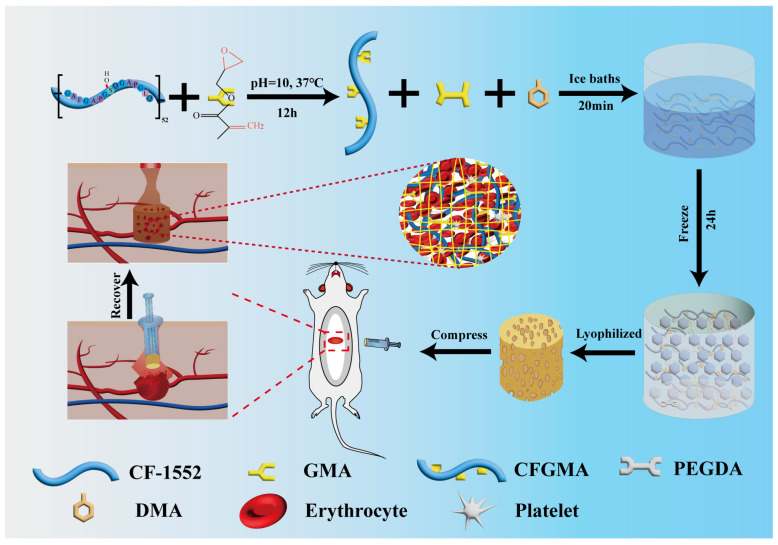
The preparation of CF/PD cryogel for non-compressible hemorrhage.

In this study, blood-triggered, rapidly shaped recoverable and biodegradable cryogels were developed for hemostasis of deep, non-compressible wounds. To achieve this, low-immunogenic recombinant collagen CF-1552 modified by glycidyl methacrylate (GMA) was employed as a biodegradable macromolecular crosslinker (CFGMA) [37,38], which cryopolymerized with poly (ethylene glycol) diacrylate (PEGDA) and dopamine methacrylate (DMA). The PBS/blood absorption rate, porosity, shape memory property, mechanical properties, adhesiveness, and degradability of the CF-1552/GMA/PEGDA/DMA (CF/PD) cryogels were systemically investigated (Scheme 1). The biocompatibility of the cryogels was characterized through hemolysis, cytotoxicity, and subcutaneous implantation tests. The coagulation ability of CF/PD was studied and investigated by erythrocyte adsorption, fibrinogen adsorption, and clinical-grade whole-blood coagulation assays. The in vivo hemostatic efficiency of the cryogel was tested using a rat liver cylindrical defect model, with comparison to a commercially available gelatin sponge. Finally, the wound repair capability of CF/PD was evaluated through wound repair experiments. This work establishes a cryopolymerization-driven strategy to engineer multifunctional cryogels, offering the transformative potential for managing non-compressible hemorrhage and facilitating wound repair.

## 2. Results and Discussion

### 2.1. Synthesis and Characterization of CFGMA and CF/PD

**Figure 1:**
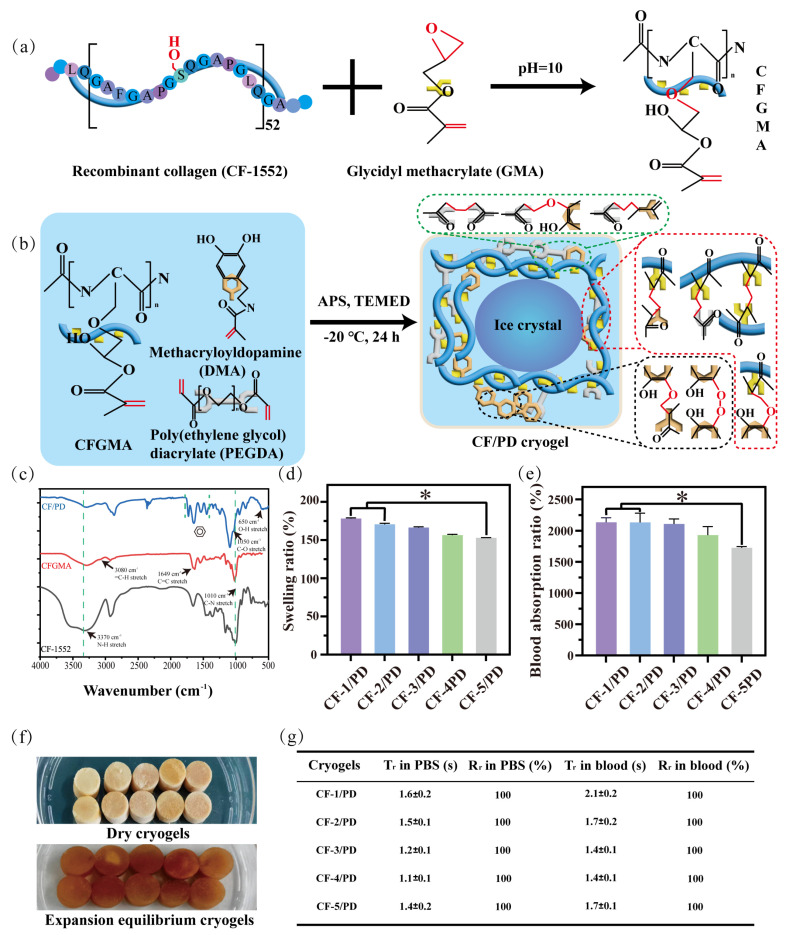
(**a**) Recombinant collagen CF-1552 modification with glycidyl methacrylate; (**b**) chemical reactions during the formation of CF/PD cryogel; (**c**) FT-IR of CF-1552, CFGMA, and CF/PD. CF/PD cryogels: (**d**) swelling ratio (*, *p* < 0.05); (**e**) PBS absorption ratio; (**f**) the dry form and the form at swelling equilibrium; (**g**) time of recovery (T_r_) and ratio of recovery (R_r_) of cryogels in PBS/blood.

CF-1552 is a recombinant collagen obtained by cycling the 15-position amino acid sequence 52 times, and CFGMA was prepared by using the hydroxyl group on the eighth serine to attack the epoxy group (Figure 1a) [39,40], which opens the ring to form an ether bond. The chemical structures of CF-1552 and modified CF-1552 (CFGMA) and modified were characterized by FT-IR spectroscopy (Figure 1c) and 1H-NMR spectroscopy (Figure S1). The successful grafting of GMA was confirmed by the appearance of a new peak representing the stretching vibration of the carbon–carbon double bond at 1649 cm^−1^ and the observation of the stretching vibration of the hydrocarbon bond on the outside of the double bond at 3080 cm^−1^ on the FT-IR spectrum. See Figure S2 for a clear FT-IR diagram. In addition, new peaks of CFGMA appeared at 5.42 ppm and 5.86 ppm on the 1H-NMR diagram, representing the stretching of two hydrogen atoms on the outside of the carbon–carbon double bond, and the peak areas were also similar. This also indicated the successful grafting of GMA. The grafting ratio of carbon–carbon double bonds was also measured by the iodine liquid method (S1.1), and the double bond grafting ratio of CFGMA was 30.3%.

CF/PD cryogel with macro-porous structure was prepared by cryopolymerization. As shown in Figure 1b, the cryogel components CFGMA, PEGDA, and DMA were homogeneously mixed in an ice bath. The radical initiator ammonium persulfate (APS) and the accelerator tetramethylethylenediamine (TEMED) were then added, and the mixture was quickly transferred to −20 °C. At low temperatures, the ice crystals slowly become larger while the cryogel components concentrate in the microphase around the ice crystals, driving the Michael reaction. After 24 h, ice crystals were removed by vacuum freeze-drying to form cryogels with macro-porous structure [19,41]. Due to its poor water solubility, DMA needs to be dissolved in the hydrophilic reagent dimethylformamide (DMF), which affects the formation of solvent ice crystals [42,43,44]; therefore, the proportion of DMA was fixed first. CF/PD cryogels are prepared by cryopolymerization, where the reaction takes place in a low-temperature environment. Thus, the initial concentrations of the three reactants were determined. In addition, in order to avoid the harmful effects of APS and TEMED, the ratio of the two was reduced as much as possible in the experiments, and the concentrations of CFGMA, PEGDA, and DMA were determined by one-way experiments (150–200 mg CFGMA, 0.2–0.6 mL PEGDA, 0–100 mg DMA). Based on the results of the one-way experiments, the CF/PD cryogel formulation was further optimized using a three-factor, three-level L_16_ (3^3^) orthogonal experiment based on the factors of shape recovery ratio (R_r_), compressive strength, and blood coagulation index (BCI) at 60 s. The results of the orthogonal experiments are shown in Tables S1 and S2.

The experimental results showed that the concentrations of CFGMA and PEGDA significantly moderated the compressive stress of the cryogels (*p* < 0.001), as variation in CFGMA and PEGDA significantly affect the solid content, crosslinking density, and hydrogen bonding of the cryogel, hence have a prominent effect on mechanical strength. DMA significantly influenced the ratio of recovery (R_r)_ and BCI (*p* < 0.001) due to the improved crosslinking and oxidative polymerization of catechol groups upon the addition of APS, which improved the elasticity of the cryogel. On the other hand, together, DMA and PEG enhance surface hydrophilicity [45,46], which not only accelerates blood absorption but also shortens the recovery time of blood-triggered shapes. The decreased BCI was also due to the aggregation and activation of erythrocytes and platelets by the catechol groups [47,48].

By constructing a regression model for the R_r_, BCI, and compressive strength, a strong linear correlation among the three was observed (R^2^ > 0.97). Model validation showed that the difference between the adjusted R^2^ and predicted R^2^ was less than 0.2, confirming that the experimental design has good predictive reliability. Through the orthogonal experiment, the optimal ratio of CFGMA, PEGDA, and DMA within 10 mL solution was determined to be 1.75% (*w*/*v*), 4% (*v*/*v*), and 0.5% (*w*/*v*), respectively. This provided a theoretical basis for constructing porous cryogel that possesses both mechanical adaptability and hemostatic efficacy. For more details, see S2.2.

The chemical structure of CF/PD cryogel was characterized by FT-IR. The result is shown in Figure 1c. Compared to CFGMA, CF/PD showed four peaks of varying intensities at 1705 cm^−1^, 1630 cm^−1^, 1456 cm^−1^, and 1341 cm^−1^, which is an important characteristic of aromatic compounds. There were new peaks at 650 cm^−1^ and 1050 cm^−1^, corresponding to hydroxide and carbon–oxygen bond stretching vibrations of the phenolic hydroxyl group. These proved the success of the grafting of the catechol moiety.

Next, more significantly, the variation of the proportion of degradable crosslinking agent of macromolecular CFGMA has a greater influence on the macro-porous structure, mechanical strength, and degradability of CF/PD cryogels. So, five CF/PD cryogels were prepared with CFGMA ratios of 1.00%, 1.25%, 1.50%, 1.75%, and 2.00% to investigate the effect of modified collagen on the properties of cryogels (Table 1). The content of CFGMA is from 1% to 2%, mainly because CFGMA content more than 2% cannot form cryogel. Figure 1f shows the dry state and swelling equilibrium of CF/PD cryogels. CF/PD cryogels were light brown when dry and brown after reaching swelling equilibrium; changes in CFGMA content had little effect on the color of the cryogel. Ultimately, the swelling and absorptivity of the cryogels were also evaluated (Figure 1d,e). All cryogels had a swelling ratio greater than 155%., confirming their robust swelling capacity that could seal deep wounds. Blood absorptions were 2126.49%, 2114.32%, 2106.50%, 1929.77%, and 1720.44%, respectively. The absorption ratio of blood was much higher than that of gelatin hemostats [49], indicating that CF/PD cryogels have good blood absorption capabilities. To summarize, CF/PD cryogels had high fluid absorption and swelling properties and, therefore, have application potential for hemostasis of non-compressive bleeding in the torso position.

### 2.2. Shape Memory Properties of the Cryogels

Cryogel is suitable for treating deep, non-compressible wounds, primarily due to its ability to quickly absorb blood to restore the original shape [23,50]. Specifically, as shown in Figure 1g, cryogels can be delivered to the bleeding site in a compressed state where they rapidly absorb blood, expand to seal the wound cavity, and apply a localized compressive force to damaged vessels [23]. Good cryogels should exhibit sufficient tissue adhesion to resist displacement by blood, thereby forming a stable physical barrier to enhance hemostatic efficiency [23,50].

The shape recovery performance of the CF/PD cryogels was evaluated in both PBS and blood (Figure 1e). In PBS, the shape recovery times (T_r_) for CF-1/PD, CF-2/PD, CF-3/PD, CF-4/PD, and CF-5/PD were (1.6 ± 0.2) s, (1.5 ± 0.1) s, (1.2 ± 0.1) s, (1.1 ± 0.1) s, and (1.4 ± 0.2) s, respectively, with all groups achieving 100% recovery ratios. The results demonstrate excellent PBS-triggered shape memory properties of the cryogels, and all formulations restored the original morphology within 2 s. In blood, the Tr for CF-1/PD, CF-2/PD, CF-3/PD, CF-4/PD, and CF-5/PD were (2.0 ± 0.2) s, (1.6 ± 0.2) s, (1.4 ± 0.1) s, (1.4 ± 0.1) s, and (1.7 ± 0.1) s, respectively, while maintaining full shape recovery (Figure 1e). The blood-triggered shape recovery of the cryogel in this study is much faster than previously reported cryogel hemostats, which are more than 40 s and 10 times slower than the PBS-triggered shape recovery due to the large viscosity of blood [17]. The fast shape recovery of the CF/PD cryogel likely originates from the synergistic interplay of material components and microstructure. The interconnected macro-porous architecture provides structural resilience for rapid shape restoration.

Meanwhile, PEGDA enhances the hydrophilicity of the cryogel, promoting blood infiltration and overbalancing the cell adhesion effect of collagen and DMA, which hinder the flow of blood. The fast shape recovery in blood guarantees fast hemostasis in real applications. Furthermore, the shape recovery property after compression for 24 h in a syringe as a delivery apparatus was examined, and the results demonstrated that both the recovery time and ratio remained stable, confirming the stability of the cryogel under prolonged mechanical stress. This stability is critical for applications such as implantable hemostatic devices and portable emergency wound dressings, ensuring reliable performance in dynamic clinical or battlefield settings.

### 2.3. Mechanical Property and Porosity of the Cryogels

**Figure 2:**
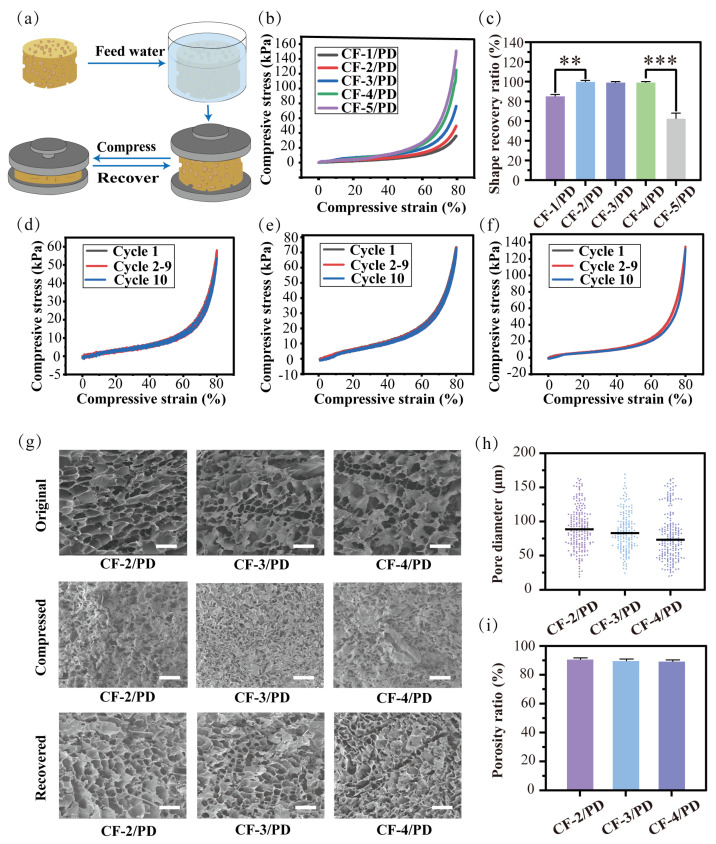
(**a**) Compression recovery cycle for the cryogels; (**b**) compression stress–strain curve at 80% compression of the cryogels; (**c**) percentage of recovery for the cryogels after 10th cyclic (**, *p* < 0.01, ***, *p* < 0.001); Compression curves at 80%: (**d**) CF−2/PD; (**e**) CF-3/PD; (**f**) CF-4/PD; (**g**) SEM results of the cryogels in its original, compressed, and recovered states, scale: 200 μm; (**h**) scatter plot of the pore size distribution of the cryogels; (**i**) porosity ratio of cryogels.

The mechanical properties of cryogels are their fundamental characteristics [51], where the modulus of elasticity is directly related to shape recovery, and the fracture toughness determines the durability of the material under cyclic compression. As shown in Figure 2a, a stress–strain experimental system was used in this study to characterize the mechanical properties of cryogels. First, as shown in Figure 2b, the stress–strain curve of the cryogel was complete without collapse in the 80% strain compression experiment, showing good mechanical properties. The compressive stress for CF-1/PD, CF-2/PD, CF-3/PD, CF-4/PD, and CF-5/PD increased with the increase in collagen content, recorded as 38 kPa, 54 kPa, 78 kPa, 134 kPa, and 153 kPa, respectively. Compared to the traditional cryogels [17,52,53,54,55], the mechanical strength of CF/PD cryogel prepared by cryopolymerization was greatly improved. Notably, all values exceeded physiological arterial blood pressure (11.3–17.3 kPa) [56,57], confirming the cryogels’ capacity to apply sufficient pressure for effective hemorrhage control.

The 80% strain cyclic compression test was performed to evaluate the durability of the CF/PD cryogels. After 10 cycles, CF-1/PD and CF-5/PD cannot return to their original shapes (Figure 2c) with noticeable collapse and deformation of the macropores; thus, they were excluded from further analysis. The cyclic compressive stress and strain curves for CF-2/PD, CF-3/PD, and CF-4/PD are shown in Figure 2d–f. There was no significant change between the compressive stress curve of the 10th cycle and that of the 1st cycle, demonstrating that CF-2/PD, CF-3/PD, and CF-4/PD cryogels possess good mechanical strength and mechanical resilience. The SEM images (Figure 2g) show that the interconnected macro-porous structures collapsed under compression but fully recovered upon fluid absorption after cyclic compression for 10 cycles.

Pore size distributions (Figure 2h) show that the average pore diameters of CF-2/PD, CF-3/PD, and CF-4/PD were 89 μm, 83 μm, and 74 μm, respectively. The average pore diameter decreased as the content of CFGMA increased due to enhanced crosslinking density. Despite this trend, all groups retained high porosity (>89 %, Figure 2i), ensuring rapid fluid uptake.

### 2.4. Biocompatibility of the Cryogels

**Figure 3:**
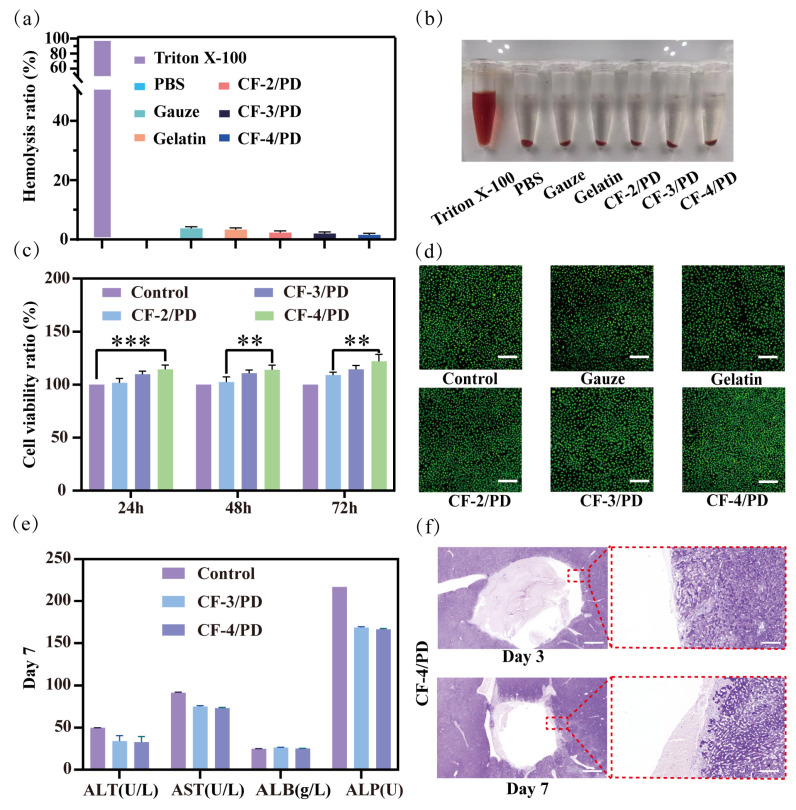
(**a**) Hemolysis ratio of the cryogels; (**b**) Sample diagram of hemolysis experiment; (**c**) Cellviability ratio of the cryogels at 24 h, 48 h, and 72 h (**, *p* < 0.01, ***, *p* < 0.001); (**d**) results of AO/EB staining of the cryogels, scale: 200 μm; liver implantation experiment in SD rats; (**e**) liver function tests on day 7; (**f**) H&E staining results of the liver implanted for 3rd and 7th day, scale: 1000 μm (**left**) 100 μm (**right**).

Deep non-compressible wounds are often located near vital organs in the trunk or in the junction area, so the cryogels for this type of wound should be biocompatible and not cause an inflammatory reaction after implantation in the traumatized person [58]. Therefore, the hemocompatibility and biosafety characteristics of the cryogel material were thoroughly investigated via three-phase validation, including blood compatibility analysis, cellular viability assessment, and animal models involving liver grafts. The results of the hemolysis experiment are shown in Figure 3a,b. Comparative analysis of experimental photographs showed that Triton X-100 induced severe erythrocyte lysis, producing a bright red supernatant due to hemoglobin release. In contrast, the supernatants of the CF-2/PD, CF-3/PD, and CF-4/PD cryogel groups were clear and colorless, indicating that the cryogels possessed good blood compatibility. The hemolysis ratio of CF-2/PD, CF-3/PD, and CF-4/PD cryogels was below 5%, with no significant change in hemolysis ratio as the proportion of CFGMA increased.

Quantification of the viability of cryogel on NCTC clone 929 (L-929) fibroblasts by leaching [17,52,59]. As shown in Figure 3c, at different time points, the cell viability ratio of the cryogel group was greater than that of the control group, indicating that the CF/PD cryogel has good cytocompatibility. After co-culturing for 24 h, the cell viability of CF-4/PD cryogel showed a significant increase compared to the control group (*p* < 0.001). Notably, the proliferation of L929 cells gradually increased with increasing CFGMA concentration. In the cell viability assay at 48 h and 72h, CF-4/PD cryogel showed a significant increase compared to CF-2/PD cryogel (*p* < 0.01). This indicates that recombinant collagen not only functions as a macromolecular crosslinking agent but also has a pro-proliferative effect on cells. This may be attributed to its own low immunogenicity advantage and activation of downstream proliferative signaling pathways through integrin–ECM interactions [60,61,62]. Consistent with quantitative results, AO/EB dual fluorescence staining (Figure 3d) showed predominantly viable (green) cells in cryogel groups, and the cell density is significantly higher than gauze and commercial gelatin sponge, further indicating the pro-proliferative effect of the cryogels.

In addition, the histocompatibility of CF-4/PD cryogel was also evaluated by examining the biomarkers for hepatic function evaluation [63]. As shown in Figure 3e, the levels of common liver function markers, including alanine aminotransferase (ALT), aspartate aminotransferase (AST), albumin (ALB), and alkaline phosphatase (ALP) on the seventh day after the hemostatic experiment were within the normal physiological range [64]. Among them, ALT and AST represent liver function, and ALP represents the metabolic function of the liver [65]. The three indices of CF-4/PD were slightly decreased compared to the normal group but still within the normal physiological range. The albumin ALB index was not significantly different from that of the normal group, indicating that the liver synthesis function of the experimental group was normal. The above results showed that CF-4/PD cryogel has good histocompatibility. Secondly, as shown in Figure 3f, the H&E staining revealed no pathological changes on the third and seventh days after CF-4/PD implantation. Collectively, the cryogels demonstrated excellent hemocompatibility, cytocompatibility, and tissue compatibility, fulfilling key criteria for clinical deployment in deep wound management.

### 2.5. In Vitro Coagulation and Mechanisms of Hemostasis of the Cryogels

**Figure 4:**
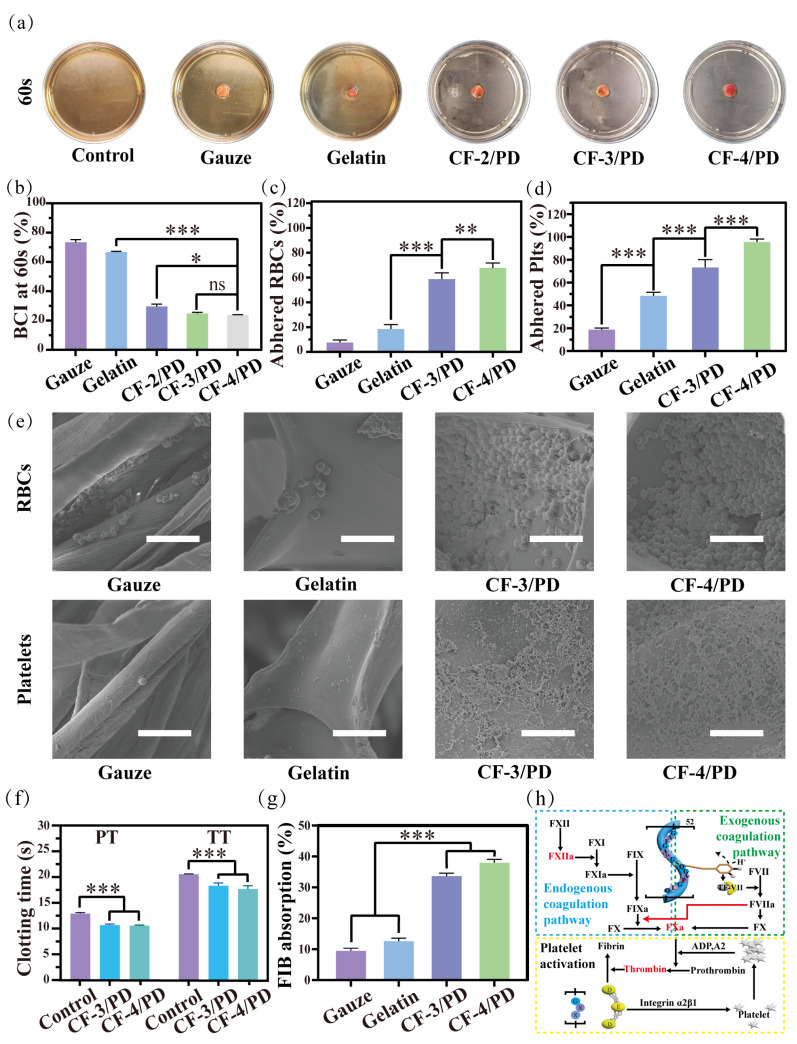
Coagulation experiment at 60s: (**a**) sample diagram; (**b**) blood coagulation index (BCI); (**c**) red blood cell adhesion; (**d**) platelet adhesion (*, *p* < 0.05, **, *p* < 0.01, ***, *p* < 0.001); (**e**) SEM of red blood cell and platelet adhesion; scale: 10 μm; (**f**) changes in prothrombin time (PT) and activated partial thromboplastin time (TT) in platelet plasma from rabbits with CF-3/PD and CF-4/PD; (**g**) fibrinogen (FIB) adsorption ratio; (**h**) schematic diagram of coagulation mechanisms of CF/PD cryogels.

The hemostatic performance of the cryogel was evaluated in vitro by the blood clotting assay (Figure 4a,b). Following scheduled incubation periods, ultrapure water was administered to lyse residual non-clotted erythrocytes, thereby facilitating spectrophotometric hemoglobin quantification to determine the material’s thrombogenic capacity.

In the coagulation experiment at 60 s (Figure 4a), there was no significant change in the color of the solution in the control, medical gauze, and gelatin groups due to the dissolution of uncoagulated blood in the deionized water after shaking. In contrast, the CF-2/PD, CF-3/PD, and CF-4/PD groups exhibited significant discoloration compared to the control group due to fast blood clotting that prevented the release of uncoagulated erythrocytes into the DI water. As shown in Figure 4b, the BCI values at 60 s for gauze, sponge, CF-2/PD, CF-3/PD, and CF-4/PD were (73.2 ± 2.6)%, (66.4 ± 0.8)%, (30.3 ± 1.8)%, (24.6 ± 0.6) %, and (23.9 ± 0.3)%, respectively. The BCI of the cryogels was significantly lower than that of the gauze and gelatin sponge (*p* < 0.001). The results demonstrate the superior hemostatic effect of the cryogels. Among the cryogel groups, CF-3/PD and CF-4/PD exhibited more efficient blood clotting than the CF-2/PD group, manifestation as significantly lower BCI values.

Mechanisms of hemostasis were investigated by quantifying erythrocyte platelet and adhesion. As shown in Figure 4c,d, the adhesion of red blood cells (RBCs) on gauze, gelatin sponge, CF-3/PD cryogel, and CF-4/PD cryogel were (19.0 ± 1.7)%, (47.3 ± 2.1)%, (72.4 ± 4.3)%, and (95.1 ± 2.3)%, respectively, demonstrating that cryogels had significantly higher erythrocyte adhesion than gauze and gelatin sponge, and the adhesion were positively correlated with the content of recombinant collagen (*p* < 0.001). The platelet adhesion exhibited a similar trend, with (7.3 ± 1.4)%, (18.2 ± 2.1)%, (59.6 ± 2.5)%, and (71.7 ± 2.2)% for gauze, gelatin, CF-3/PD, and CF-4/PD, respectively. SEM images (Figure 4e) reveal only a small number of red blood cells with biconcave disc shape on gauze and the gelatin sponge. In contrast, a significantly higher number of red blood cells in aggregated states were adhered in the macro-porous structures of the CF-3/PD and CF-4/PD. Similarly, the SEM images of platelets showed a large number of platelets in activated states adhered in the macro-porous structures of the cryogels, while only a small number of platelets were adhered to the surface of the control groups.

In addition, the activation of coagulation factors plays a crucial role in the hemostatic process. The coagulation mechanism of CF/PD was studied by measuring prothrombin time (TT) and thromboplastin time (PT). As shown in Figure 4f, revealed CF/PD cryogels achieved accelerated thrombin time parameters (*p* < 0.001) versus controls. Suggesting that it is consistent with the modulation of the hemostatic response by collagen derivatives initiated by contact activation of the intrinsic coagulation pathway through an FXII-mediated cascade [66]. In addition, the thromboplastin time (PT) of the CF/PD cryogels was significantly shorter than that of the control group (*p* < 0.001), suggesting that the CF/PD cryogel also activated the extrinsic coagulation pathway. The phenomenon may arise from reversible oxidation of the catechol moiety to produce semiquinone or quinone structures that mimic the function of tissue factor (TF), forming a complex (TF-VIIa) with coagulation factor VII and initiating the external coagulation cascade [67].

Finally, the fibrinogen (FIB) adsorption of CF-3/PD and CF-4/PD (Figure 4g) were (34.7 ± 1.2)% and (37.8 ± 1.4)%, respectively. The adsorption of CF/PD cryogels was significantly higher than that of gauze and gelatin sponges (*p* > 0.001). The results confirmed that cryogels can rapidly absorb fibrinogen from the blood [68] and create favorable conditions for secondary hemostasis (fibrin clot formation at the thrombus site) [69].

In summary, the hemostatic mechanism of CF/PD cryogel is discussed here in Figure 4h. First, CF/PD cryogel is made with a large molecule of modified recombinant collagen as a macro-porous structural scaffold to activate the endogenous coagulation pathway through contact activation of coagulation factor XII (FXII) [66]. The semiquinone or quinone structures on the surface of the cryogel mimic the function of TF to bind FVII and activate the exogenous coagulation pathway [67]. Whereas the exogenous coagulation pathway is much faster than the endogenous coagulation pathway, the generated coagulation factor IIa (FIIa) has a positive feedback effect on the endogenous coagulation pathway. In conclusion, after CF/PD cryogel contact with blood, it accelerates thrombin formation by activating endogenous and exogenous coagulation pathways [70,71]. At the same time, the GXX sequence on the recombinant collagen complex binds to the D structural domain of fibrinogen, promoting fibrinogen adsorption and platelet aggregation [72]. Finally, fibrinogen is converted to fibrinogen, which forms a fibrin network in the presence of thrombin, netting white and red blood cells and forming blood clots.

### 2.6. In Vivo Hemostasis of the Cryogels

**Figure 5:**
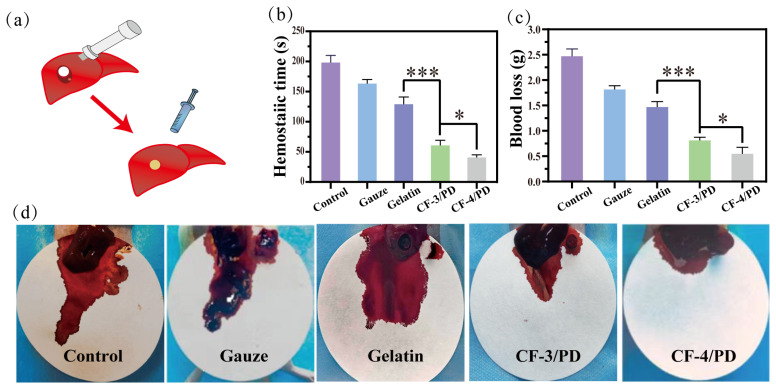
Rat liver cylindrical injury model: (**a**) experimental schematic diagram; (**b**) hemostasis time; (**c**) blood loss volume (*, *p* < 0.05, ***, *p* < 0.001); (**d**) photograph of material removal after hemostasis in a rat liver cylindrical defect model.

The hemostatic capacity of cryogels for non-compressive hemorrhage was assessed using a rat liver cylindrical (8 mm) defect model (Figure 5a). The hemostatic time (Figure 5b) for control, medical gauze, commercial gelatin sponge, CF-3/PD and CF-4/PD groups was (191 ± 17) s, (163 ± 13) s, (129 ± 15) s, (60 ± 11) s, and (49 ± 5) s, respectively, which showed a gradient decrease. Compared to medical gauze and gelatin sponge, CF/PD cryogels showed a significant reduction in hemostasis time (*p* < 0.001). Moreover, CF-4/PD results in faster hemostasis compared to CF-3/PD due to the presence of higher recombinant collagen content. Compared to the previous research [17], CF/PD cryogel further reduced the hemostatic time to less than one minute, showing a superior hemostatic effect. The results of blood loss (Figure 5c) for control, medical gauze, commercial gelatin sponge, CF-3/PD, and CF-4/PD groups were (2.47 ± 0.38) g, (1.87 ± 0.24) g, (1.47 ± 0.30) g, (0.84 ± 0.21) g, and (0.64 ± 0.17) g, respectively. Compared to medical gauze and gelatin sponge, CF/PD cryogels showed significant decreases in blood loss (*p* < 0.001). Among them, CF-4/PD demonstrates the smallest blood loss. Visual inspection of the blood loss (Figure 5d) further confirmed that CF-4/PD has the best in vivo hemostatic effect.

Control of non-compressible hemorrhage is achieved through three main dimensions. First, with the aid of a syringe, the compressed cryogel can be injected into narrow and deep wounds, expanding rapidly on contact with blood to form a physical barrier to seal the wound. In addition, the tissue adhesion capacity brought about by catechol can better form a physical barrier and accelerate hemostasis. Second, the hydrophilicity and high fluid absorption of the cryogel allow for rapid absorption of blood and enrichment of blood cells through physical retention and blood cell affinity (catechol and recombinant collagen). Last but not least, collagen specifically binds to platelet membrane surface receptors, further promoting platelet activation, accelerating thrombin generation through the intrinsic coagulation pathway, and adsorbing fibrinogen to promote thrombus formation [72,73,74].

### 2.7. Wound Healing Study of the Cryogels

**Figure 6:**
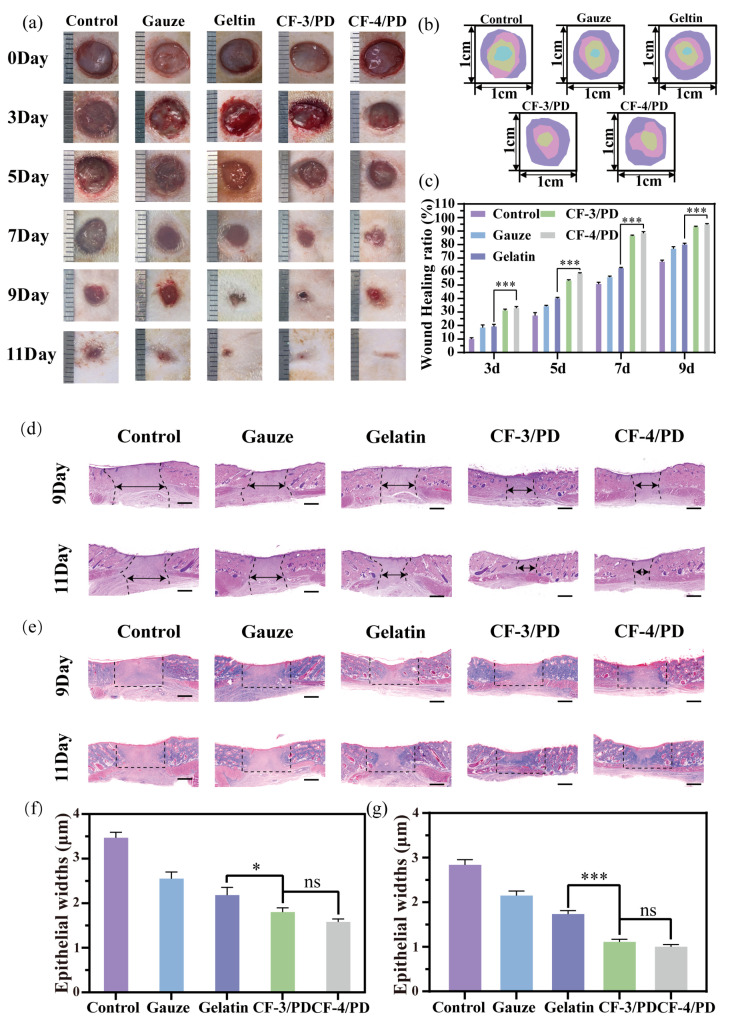
(**a**) Wound healing experimental diagram; (**b**) wound shrinkage indentation plots; (**c**) wound area shrinkage graph; (**d**) H&E staining plots; (**e**) Masson staining; the scale bars in (**d**) and (**e**): 1000 μm. Statistical results of epithelial widths in the skin wound healing model for the control, gauze, gelatin, CF-3/PD, CF-4/PD: (**f**) 7th day; (**g**) 11th day (*, *p* < 0.05, ***, *p* < 0.001).

The interconnected macro-porous architecture of CF/PD cryogels facilitates nutrient exchange and integrin/catechol-mediated cell attachment [75], acting as a scaffold to support cell migration, cell proliferation, and wound healing [76,77]. The wound healing effects of CF/PD cryogels were investigated in a rat dorsal full-layer defect model. At each time point (Figure 6a–c), CF-3/PD and CF-4/PD have significantly faster wound closure than the control, medical gauze, and gelatin sponge groups (*p* < 0.001). After 11 days post-treatment, the wounds in the cryogel groups achieved near-complete wound closure, whereas the wounds in the blank gauze and gelatin sponge groups were not completely closed (Figure 6a). The results demonstrated that cryogels had a good wound repair effect and that CFGMA played an important role in wound repair.

The quality of skin regeneration and collagen deposition at the wound site was assessed by H&E and Masson staining. H&E results for the ninth day of wound treatment (Figure 6d,f) showed that the epithelial widths of CF-3/PD and CF-4/PD were (1.80 ± 0.10) mm and (1.58 ± 0.08) mm, respectively, and were significant compared that of commercially available gelatin. Staining results and epithelial width on the 11th day (Figure 6g) were similar to those on the 9th day. In addition, Masson results (Figure 6e) are consistent with H&E by the 11th day. Masson’s trichrome staining demonstrated denser collagen deposition and thicker connective tissue in cryogel groups, indicating advanced tissue remodeling. All of these results indicate that CF/PD cryogels have good wound repair function, highlighting their excellent potential as a hemostatic material. The possible reasons are as follows. First of all, cryogel absorbs exudate when applied to the wound and provides a sterile, breathable environment [78,79]. In addition, recombinant collagen-based macro-porous cryogels function as a scaffold that provides high specific area and integrin binding sites to induce cell migration, proliferation, and differentiation [80,81,82].

### 2.8. Degradability of the Cryogels

**Figure 7:**
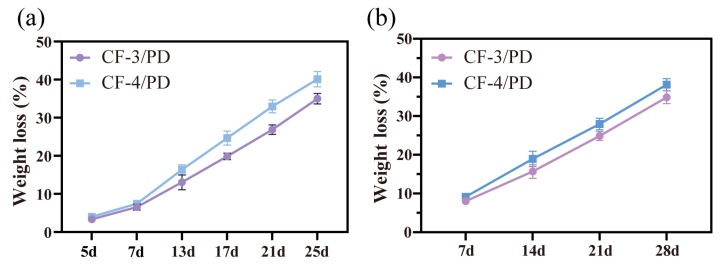
(**a**) In vitro degradation; (**b**) in vivo degradation.

Most cryogels are not designed for hemostasis and typically lack biodegradability [21,22,83,84,85]. Hemostatic materials for deep and penetrating wounds are inherently difficult to remove and are prone to cause secondary injuries to the traumatized person during the removal process. Therefore, biodegradability emerges as a critical requirement for such materials. In this study, in vitro degradation and subcutaneous implantation experiments in SD rats were performed to evaluate the degradability of cryogels. As shown in Figure 7a,b, on day 25 of the in vitro degradation experiments, the weight loss of CF-3/PD and CF-4/PD were (35.5 ± 1.7)% and (40.4 ± 2.1)%, respectively. On day 28 of subcutaneous degradation, the degradation ratios of CF-3/PD and CF-4/PD were (34.4 ± 1.9)% and (38.2 ± 2.4)%, respectively. The weight losses of CF/PD cryogels showed a linear correlation with time, indicating that the cryogels were biodegradable.

## 3. Conclusions

In this study, cryogel based on methacrylated recombinant collagen CF-1552, PEGDA, and DMA was developed by cryopolymerization for hemostasis of non-compressive wounds. The CF/PD cryogel exhibits 1.5 s blood-triggered shape recovery, tissue adhesiveness, antibacterial properties, and enhanced mechanical resilience. Compared to commercial gelatin sponges, CF/PD exhibited superior hemostatic performance, shortening clotting time by 74.4% and reducing blood loss by 76.5% in a rat liver defect model, attributing to the following mechanisms: (1) physical compression hemostasis due to fast shape recovery and mechanical strength; and (2) accelerated clot formation through platelet activation, fibrinogen adsorption, and dual intrinsic/extrinsic coagulation pathway modulation. Moreover, CF/PD cryogel has biodegradability and promotes wound repair. Therefore, CF/PD cryogels show great potential for both hemostasis and wound healing in deep, non-compressible wounds.

## 4. Materials and Methods

### 4.1. Materials

CF-1552 (China patent number: ZL01106757.8, Mr = 97000) was purchased from Xi’an JUZI Biology Gene Technology Co., Ltd., (Xi’an, China). Glycidyl methacrylate (GMA, purity >99.5%), poly (ethylene glycol) acrylate [PEGDA, molecular weight (MW): 700, purity > 98%], dialysis bag [molecular weight cut off (MWCO)8000–12,000], and 3-methacryloyl dopamine (DMA, purity > 97%) were purchased from Shanghai Macklin Co., (Xi’an, China). N,N-dimethylformamide (DMF, purity > 99.5%) was purchased from Tianjin Fuyu Chemical Co., (Tianjin, China). Phosphate Buffer Saline (PBS, pH = 7.4), sodium hydroxide (NaOH, purity > 99.5%), and ammonium persulfate (APS, purity > 98%) were purchased from Tianjin Damao Chemical Reagent Partnership Enterprise (Tianjin, China). Tetramethylethylenediamine (TEMED, purity > 98%) was purchased from Shanghai Zhanyun Chemical Co., Ltd., (Shanghai, China).

### 4.2. Synthesis of CFGMA and CF/PD

CFGMA was synthesized using a previously reported method [86,87]. Briefly, CF-1552 (1.2 g) was dissolved in a solution of DMF/ultrapure water (1:2; *v*/*v*, 120 mL), and GMA (3.6 mL) was added slowly dropwise at 400 r/min. The solution pH was adjusted to 9 by adding NaOH (1 M), and then the mixture was stirred and reacted at 400 r/min in a 40 °C water bath for 12 h. The pH was measured every hour, and the pH of the mixture was adjusted to 9 by NaOH (1 M). The reaction solution was dialyzed in deionized water for 5 d and lyophilized to obtain the final product.

CFGMA/PEGDA/DMA (CF/PD) was also synthesized following a modified protocol from prior studies [17,86]. First, the obtained CFGMA (100 mg) under constant stirring in ultrapure water (9.55 mL), followed by the addition of PEGDA (0.4 mL), was added at 500 r/min. Then DMA (50 mg) dissolved in DMF (0.05 mL) was added to the mixture together with the activator APS (0.1 g), and the liquid was purged with N2 for 15 min. The mixture was then sealed and placed in an ice bath for slow cooling to freezing point (20 min), and the redox agent TEMED (10 µL) was added. Finally, the mixture was transferred to a centrifuge tube and frozen at −20 °C for 24 h. In order to avoid the toxicity of the initiator, accelerator, and unreacted monomers, the cryogels were washed with deionized water 10 times and freeze-dried.

The optimization of the three components of the CF/PD cryogel was performed using a three-level L_16_ (3^3^) orthogonal experimental design (details in Supporting Information). The detailed experimental design is shown in Table 1.

### 4.3. Characterization of CFGMA and CF/PD

The chemical structure of CFGMA was analyzed by FT-IR spectroscopy. The successful grafting of GMA was verified by ^1^H-NMR (400 MHz, D_2_O). In addition, the unsaturation degree of CFGMA and the grafting ratio of carbon–carbon double bonds were determined by the iodine solution method. See S1.1 for details.

The structural formula of CF/PD was observed by FT-IR with ^1^H-NMR (400 MHz, D_2_O). Refer to Supporting Information for detailed methods.

### 4.4. Determination of Shape Recovery of Cryogels in Blood or Tissue Fluid

The cryogels were prepared as cylinders with a height of 10 mm and a diameter of 10 mm. The cryogel was compressed in a 1mL syringe against PBS/blood, and then the cold gel was rapidly pushed out, and the shape recovery time and degree of shape recovery of the cryogel were observed and recorded. The cryogel was compressed in a 1mL syringe against PBS/blood, and then the cryogel was rapidly pushed out, and the shape recovery time and degree of shape recovery of the cryogel were observed and recorded. Calculate the ratio of recovery of the blood-sucking shape of the cryogel according to the following formula [17,88]:

(1) $$\mathrm{Shape}\;\mathrm{recovery}\;\mathrm{ratio}\;(\%)=\frac{{\left(\frac{D}{2}\right)}^{2}\times 3.14\times H}{v}\times 100\%$$

where *D* represents the recovered diameter of the cryogels, *H* represents the recovered height of the cryogels, and *v* is the volume of the cryogel (height 10 mm, diameter 10 mm).

In addition, the initial, compressed, and recovered states of the cryogel were observed using scanning electron microscopy to evaluate the shape recovery performance of the cryogel in blood or tissue fluids.

### 4.5. Characterization of the Physical Properties of Cryogels

The physical properties of each group of CF/PD cryogels were evaluated through the maximum absorption capacity by PBS, swelling ratio, porosity, scanning electron microscopy (SEM), and pore size analysis [88,89,90]. For details, please refer to Supporting Information.

Then the mechanical properties and durability of the swelling equilibrium cryogels were tested using a universal testing machine (Shanghai, China) [17,90]. The cryogel was first compressed to 80% strain at 2 mm/min, and the compressive stress–strain curve was recorded. The process was then cycled 10 times at 10 mm/min to observe the shape recovery of the cryogel and to record the compressive stress–strain curve.

### 4.6. Biocompatibility Assay of Cryogels

Based on previous methods in the literature [91], the blood compatibility, cell compatibility, and tissue compatibility of each group of CF/PD cryogels were evaluated through hemolysis tests, cell compatibility tests, subcutaneous implantation experiments in rats, and liver regeneration experiments. At the same time, the in vivo biodegradation profile of cryogels was further characterized using a rat subcutaneous implantation model. See Supporting Information for details.

### 4.7. In Vitro Hemostatic Performance of Cryogels

Collection of blood and preparation of blood-related components as detailed in Supporting Information.

The cryogels were prepared to a height of 5 mm and a diameter of 8 mm, placed on petri dishes, and preheated in an oven (37 °C) for 30 min. Then 100 µL of citrated rabbit blood was added to 10 µL of calcium chloride (0.2% M), shaken, and quickly added dropwise to the cryogel surface, and incubated at 37 °C for 60 s. Finally, 25 mL of deionized water was added and shaken at 150 r/min for 5 min on a shaker to fully lyse the unadsorbed erythrocytes. Absorbance (ODsample) was measured at 540 nm by taking 100 µL of supernatant. Blank control was the absorbance (OD reference value) of the supernatant of 100 µL rabbit blood with citric acid added with 10 µL of calcium chloride (0.2% M) in 25 mL of deionized water. The control group was medical gauze and commercially available gelatin sponges. Each group was repeated three times, and the Blood Clotting Index (BCI) of the cryogels was calculated according to the following formula [90]:

(2) $$\mathrm{BCI}\;(\%)=\frac{OD_{\mathrm{sample}}}{OD_{0\mathrm{reference}\;\mathrm{value}}}\times 100\%$$

### 4.8. Hemostatic Capacity and Hemostasis Mechanism Analysis of Cryogels

The coagulation function of CF/PD cryogels was evaluated by erythrocyte platelet adhesion, fibrinogen adhesion, and clinically standardized coagulation assays according to previous methods in the literature [51,88,92]. Mechanisms of hemostasis in CF/PD are explored and discussed. See Supporting Information for details.

### 4.9. In Vivo Hemostasis of Cryogels

All animal procedures were approved by the Northwest University Laboratory Animal Care and Ethics Committee (NWU-AWC-20240734R).

In vivo hemostatic properties of cryogels were evaluated by a cylindrical (d: 8 mm) liver defect model in SD rats [90]. The experimental groups consisted of 5 SD rats (male, 250–300 g) per group. See Supporting Information for details.

### 4.10. Wound Healing of Cryogels

The pro-wound-repair properties of CF/PD cryogels were evaluated by a model of total dorsal defects in SD rats according to previous methods in the literature [17,18]. See Supporting Information for details.

We assessed the inflammatory response and collagen deposition in the wound by hematoxylin–eosin (H&E) staining and Masson’s staining.

### 4.11. Statistical Analysis

All experiments were performed with 3 or more independent replications. The experimental data were expressed as mean (Means) ± standard deviation (SD). Scientific tests were used for all experimental data. * *p* < 0.05 was considered statistically significant.

### 4.12. Statistics and Reproducibility

All tests were processed in triplicate and similar results were acquired. Each group has three independent samples. Statistical ana-lyses were performed using GraphPad Prism 8 software. Values are expressed as the means ± standard deviation (SD). Comparison between two groups was per-formed by unpaired two-tailed *t*-test. For multiple group comparison, one-way ANOVA with Tukey’s multiple comparison test was used. * *p* < 0.05 was considered to be statistically significant.

## Figures and Tables

**Table 1:** Specific composition of different cryogels.

Cryogel	CFGMA (%, *w*/*v*)	PEGDA (%, *v*/*v*)	DMA (%, *w*/*v*)
CF-1/PD	1.00	4.0	0.50
CF-2/PD	1.25	4.0	0.50
CF-3/PD	1.50	4.0	0.50
CF-4/PD	1.75	4.0	0.50
CF-5/PD	2.00	4.0	0.50

## Data Availability

The original contributions presented in this study are included in the article/Supplementary Material. Further inquiries can be directed to the corresponding authors.

## Supplementary Materials

The following supporting information can be downloaded at https://www.mdpi.com/article/10.3390/gels11060445/s1, Figure S1. (a) 1H-NMR patterns of CFGMA and CF-1552. (b) UV curing study. Table S1. Orthogonal experimental results. Table S2 Analysis of the range and significance of orthogonal experimental results. Figure S2. FT-IR mapping of CF-1552, CFGMA and CF/PD. Figure S3. Results of antimicrobial experiments by groups (CF/P is the experimental group without DMA). Video S1, PBS absorption. Video S2, Blood absorption. Video S3, Rat liver hemostasis (1 min).
