# Supplementary material for: Biodegradable and Mechanically Resilient Recombinant Collagen/PEG/Catechol Cryogel Hemostat for Deep Non-Compressible Hemorrhage and Wound Healing
Source: Gels. 2025 Jun 10;11(6):445. doi: 10.3390/gels11060445 (PMC12192300; doi:10.3390/gels11060445)
Supplement: Supplementary file 1 [file gels-11-00445-s001.zip › gels-3661159-supplementary.pdf]

Supporting Information

Biodegradable and mechanically resilient recombinant collagen/PEG/catechol cryogel hemostat for deep noncompressible hemorrhage and wound healing

Yuanzhe Zhang [a,b,c], Tianyu Yao [a,b,c], Ru Xu [a,b,c,d], Pei Ma [a,b,c], Jing Zhao* [a,b,c], Yu Mi* [a,b,c]

a Engineering Research Center of Western Resource Innovation Medicine Green Manufacturing, Ministry of Education, School of Chemical Engineering, Northwest University, Xi'an, 710127, China
b Shaanxi Key Laboratory of Biomaterials and Synthetic Biology, Shaanxi R&D Center of Biomaterials and Fermentation Engineering, School of Chemical Engineering, Northwest University, Xi'an, 710127, China
c Biotech. & Biomed. Research Institute, Northwest University, Xi'an, 710127, China
d Xi'an Giant Biogene Technology Co.,Ltd., Xi'an 710069, China
†: These authors contributed equally to this work. e-mail: J. Z. zhaojing@nwu.edu.cn; Y. M. mi_yu@nwu.edu.cn. Tel: J. Z. 18066523386; Y.M. 13572539005.

S1. Experimental Section

S1.1. The grafting ratio of the double bonds in CFGMA

The carbon-carbon double bond grafting ratio of CFGMA was determined by iodometric titration according to the available literature treatments. Specifically: Configure the Wei-type reagent according to the “National Standard” of Chinese (Guo Biao 676): dissolve 1.9 g of iodine chloride in 100 mL of glacial acetic acid. An amount of CFGMA was weighed into an iodine measuring flask and 6 mL of trichloromethane was added to completely dissolve the reagent. Add 1 mL of Wei-style reagent, mix thoroughly and store at room temperature away from light for 2 h. Remove the mixture and add 2 mL of Wade's Reagent and a quantity of deionized water. This was followed by titration with a standard titrant of sodium thiosulfate until the yellow color disappeared. Add 2 mL of starch indicator and proceed to titrate until the blue color disappears and record the volume of titrant. A separate set was made without CFGMA as a blank control. The following formula was used to calculate the double bond content and grafting ratio:

$$Y_1\ (\text{mmoL/g}) = \frac{c \times (V_1 - V_0)}{m} \quad (S1)$$

$$\text{Grafting ratio}\ (\%) = \frac{Y_1}{Y_0} \times 100\% \quad (S2)$$

Where Y_1 represents the measured unsaturation value (double bond content), c represents the concentration of sodium thiosulfate titrant used, V_1 the volume of titrant used for the actual sample, V_0 the volume of titrant used for the actual blank, and Y_0 the unsaturation value (double bond content) of the theoretical 100 % grafted CFGMA.

S1.2. Characterization of the physical properties of cryogels

S1.2.1. Determination of maximum liquid absorption ratio of cryogels

The cryogel was weighed (*W_0*) and immersed in PBS and blood, respectively. After waiting for two hours and removing it, the liquid on the surface of the hemostatic sponge was wiped off with filter paper and weighed (*W_1*). The liquid absorption ratio of the hemostatic sponge was calculated according to the following formula:

$$\text{Liquid Absorption ratio (\%)} = \frac{W_1}{W_0} \times 100\% \tag{S3}$$

The pH of PBS was neutral and the blood was fresh citrate anticoagulated rabbit blood.

S1.2.2. Porosity of cryogels

The porosity of CF/PD cryogels were determined by liquid displacement method.CF/PD cryogels were weighed (W_1) and then submerged in anhydrous ethanol for 10 h. After the crystalline gel absorbed full of ethanol, it was removed, and the ethanol was wiped off the surface of the crystalline gel with filter paper and weighed (W_0). The porosity of the cryogels were calculated according to the following equation:

$$\text{Porosity ratio (\%)} = \frac{W_1 - W_0}{pV} \times 100\% \tag{S4}$$

Where V is the volume of CF/PD cryogels and p is the density of anhydrous ethanol.

S1.2.3. Dilatationof cryogels

The CF/PD cryogels was cut into cylinders with height of 10 mm and diameter of 10 mm, immersed in PBS, and left for 30 min to observe and measure the height and diameter of the cryogels after expansion. The expansion ratio of cryogels is calculated according to the following formula:

$$\text{Expansion ratio (\%)} = \frac{H_1}{H_0} * \left(\frac{D_1}{D_0}\right)^2 * 100\% \tag{S5}$$

Where H_1 is the height of the CF/PD cryogel after expansion and H_0 is the height of the CF/PD before expansion. d_1 is the radius of the CF/PD after expansion and d_0 is the radius of the CF/PD before expansion.

S1.3. Measurement of mechanical strength of cryogels

Dry cryogels (height 10 mm, diameter 8 mm) were compressed to 20% of their original height (height 2 mm) with a universal tension machine at a rate of 1 mm/min. The compressive stress-compressive strain curves of the hemostatic sponges were recorded by instrumentation. The compressive stress-compressive strain curves of the hemostatic sponges in the wet state were the same as those of the dry hemostatic sponges, and the dry hemostatic sponges were replaced by dissolution-equilibrated hemostatic sponges (height 10 mm, diameter 10 mm).

For cyclic compression experiments, a dissolution-equilibrated Dry cryogels (height 10 mm, diameter 8 mm) were compressed to 20% of their original height (height 2 mm) and cycled 10 times. A small amount of deionized water was dropped on the test bench before the experiment. Data were recorded and compressive stress-compressive strain curves were

plotted for the dissolved-equilibrium condition. Cycles 1, 2-9 and 10 were labeled to observe the mechanical stability of the cryogels.

S1.4. Biocompatibility of cryogels

S1.4.1. Cytocompatibility assay of cryogels

The cytocompatibility of CF/PD crystalline gel was assessed by a generalized MTT assay. First, cell culture medium (RPMI-1640+10 % fetal bovine serum+1 % penicillin/streptomycin mixture) was prepared. The Co60-sterilized CF/PD cryogels were submerged in the prepared cell culture medium at ratio of 1:10 and placed at 37 ℃ for 48 h to obtain mixed cell culture solution for standby. The passaged L929 cells were counted through counting plate and then inoculated into a 96-well plate at 1000 cells per well.100 μL of cell culture medium was first added and incubated in a cell culture incubator for 24 h. The cells were then cultured in a cell culture incubator. The cell culture solution was then aspirated, mixed cell culture solution was added, and incubation was continued for 24 h, 48 h, and 72 h in the cell culture incubator. Finally, in chronological order, 50 μL of MTT solution was added to each well and the cells were incubated for 120 min in the cell culture incubator. At the end of the incubation, the mixed solution was removed and 150 μL of DMSO solution was added. The absorbance values (490 nm) of individual wells of the 96-well plate were measured by an enzyme marker and recorded as OD_{sample}. Cell cultures were used as controls at each time point, and the absorbance value of the control was used as the $OD_{negative}$. The cytocompatibility of CF/PD cryogels was calculated according to the following equation:

$$\text{Cell viability ratio (\%)} = \frac{OD_{sample}}{OD_{negative}} \times 100\% \tag{S6}$$

Then the experiment was repeated as above, and the cells were cultured for 24 h. The cells were stained using the AO/EB staining kit according to the instructions. Finally, the living and dead staining structures of the cells were observed with a fluorescence microscope under dim light.

S1.4.2. Hematocompatibility measurement of cryogels

The cryogels were pulverized into powder using a pulverizer and then dispersed in PBS to make a suspension. After preheating the suspension in 37 °C water bath for 30 min, 0.5 mL of the erythrocyte suspension was added to the samples (0.5 mL) and incubated for 1 h at 37 °C, followed by centrifugation at 1500 rpm for 10 min. The absorbance of the supernatant was measured at 540 nm (OD_{sample}). 0.5 mL of erythrocyte suspension was added to 0.5 mL of 0.1% Triton X-100 and 0.5 mL of PBS to serve as a positive control and a negative control, respectively. The blood compatibility of CF/PD cryogels was calculated by the following equation:

$$\text{Hemolysis ratio (\%)} = \frac{OD_{sample} - OD_{negative\ control}}{OD_{positive\ control} - OD_{negative\ control}} \times 100\% \tag{S7}$$

S1.4.3 Histocompatibility of cryogels

In the same rat liver cylindrical defect model, CF/PD cryogel was implanted into the rat

liver cylindrical defect wound after hemostasis surgery, pressed back into the rat abdominal cavity, sterilized and sutured the wound. The survival ratio of the rats was observed, and two rats were killed at 7th day and 14th day of survival, and the major liver functions of the rats were characterized according to the requirements of the kits (alanine aminotransferase ALT assay kit, aspartate aminotransferase AST assay kit, albumin assay kit, and alkaline phosphatase assay kit). And hematoxylin-eosin (H&E) staining was performed on the defective liver to observe the histocompatibility of crystalline gum.

S1.5 Antimicrobial Testing

The LB solution was assembled in a 500 mL conical flask (500 mL of ultrapure water + 10 g of peptone + 5 g of yeast powder + 10 g of agar: (2 bottles; 100 mL ultrapure water + 2 g peptone +1 g yeast powder). After autoclaving, it is made into solid medium and liquid medium. Sterilized dopamine-containing and dopamine-free materials were used for co-culture with Staphylococcus aureus and Escherichia coli, and diluted cultures were spread on the plates using the smear plate method. Observe the number of colonies on the plate.

S1.6 Hemostatic mechanisms of cryogels

S1.6.1 Preparation of blood and blood-related components

Whole blood (CWB) was collected using sodium citrate blood collection tubes (2-5 mL, 1:4 ratio of blood to 3.2 % sodium citrate) from a vein behind the ear of New Zealand White rabbits that had been housed for at least one week (maximum one month). The collected CWB was centrifuged on a centrifuge at 1000 rpm for 15 minutes to obtain a stratified solution. One of the supernatants was platelet-rich plasma (PRP), which was removed with a rubber-tipped pipette. The lower dark red precipitate was mixed with PBS to obtain red blood cell suspension (RBC). The CWB was placed on a centrifuge and centrifuged at 3500 rpm for 15 min, the solution was clearly stratified and the supernatant was platelet poor plasma (PPP).

S1.6.2 Erythrocyte and platelet adsorption of cryogels

The CF/PD cryogels were made into a cylinder with height of 5 mm and diameter of 8 mm using a hole punch, and 200 μL of CWB was added dropwise to the cryogels after preheating through 37 °C oven. Incubate in 37 °C water bath for 60 min and remove unadhered erythrocytes from the surface by washing with PBS. The samples were then submerged in 5 mL of deionized water and the unadhered erythrocytes were lysed to release hemoglobin. After 60 min, 150 μL of supernatant was taken separately by five-point sampling method to measure the absorbance (540 nm) recorded as OD_{sample}. The absorbance value of the same treatment (200 μL RBC, 5 mL deionized water) was used as a control and recorded as $OD_{reference\ value}$. Medical gauze and commercially available gelatin sponges were used as controls. The erythrocyte adsorption value of cryogels were calculated according to the following equation:

$$\text{Percent of adhered RBCs (\%)} = \frac{OD_{\text{sample}}}{OD_{\text{oreference value}}} \times 100\% \quad \text{(S8)}$$

In addition, sponges washed with PBS were fixed in electron microscope fixative for 120 min. Finally, gradient dehydration with gradient ethanol (10 %, 20 %, 30 %, 40 %, 50 %, 60 %,

70 %, 80 %, 90 %, 100 %) was performed and the number and morphology of adherent erythrocytes were observed by SEM after drying at room temperature.

As in the erythrocyte adsorption assay, 500 μL of PRP was added dropwise to the surface of CF/PD cryogels after preheating the cryogels. After a water bath at 37 °C for 60 min, the cryogels were submerged in 5 mL of PBS to wash the unadhered platelets. The cryogels were then submerged in 1% Triton X-100 solution to cleave to release LDH. Allowed to lyse for 60 min and processed according to the LDH kit instructions to determine the released LDH. The absorbance of the supernatant was measured (440 nm) and the LDH value was calculated and recorded as OD_{sample}. The absorbance of the blank control group with the same treatment was used to obtain the LDH value recorded as $OD_{reference\ value}$. Medical gauze and commercially available gelatin sponges were also used as synchronized treatments as a control. Each group was repeated three times and the percentage of platelets adhered to the cryogels was calculated according to the following formula:

$$\text{Percent of adhered platelets ratio (\%)} = \frac{OD_{\text{sample}}}{OD_{\text{oreference value}}} \times 100\% \quad \text{(S9)}$$

As with the erythrocyte adhesion assay treatments, dehydration was carried out using gradient ethanol, followed by drying. The number of platelet adhesions and the morphology of platelets were observed by SEM.

S1.6.3 Fibrinogen adsorption of cryogels

Prepare 10 mL of neutral PBS for heating in 37 °C water bath to set aside. A quantity of bovine fibrinogen (FIB) was taken and dissolved in 10 mL PBS. 10 mg of CF/PD cryogels were submerged in 2 mL of PBS for 30 min. The sample was then removed and 2 mL of the FIB solution was added dropwise to the surface of the solubilized equilibrated CF/PD cryogels. Subsequently, the prepared samples were shaken in thermostatic shaker for 120 min (60 rpm). At the end of shaking, the cryogels were compressed to squeeze out the solution within the cryogels and the absorbance of the solution was measured (280 nm), and the FIB concentration was calculated as the residual FIB concentration , which was recorded as OD_{sample}. The same treated medical gauze and commercially available gelatin sponges were used as controls. The dissolved 1 mg/mL FIB solution was recorded as $OD_{reference\ value}$ as reference value. The percentage of fibrinogen adsorbed by cryogels was calculated according to the following formula:

$$\text{Percent of absorbed fibrinogen ratio (\%)} = \frac{OD_{reference\ value} - OD_{sample}}{OD_{reference\ value}} \times 100\% \quad \text{(S10)}$$

S1.6.4 Clinical standard coagulation of cryogels

The prothrombin time (PT) and activated partial thromboplastin time (APTT) of CF/PD crystalline gels were measured by fully automated coagulation analyzer (RAC-030, China). First for PT, 500 μL of PPP was taken with 10 mg of sample and preheated in a 37 ℃ oven for 5 min, followed by mixing the two with PT reagent to measure the PT value. For APTT, 500 μL

of prepared PPP was taken and mixed with APTT reagent and preheated for 5 min, followed by the addition of 10 mg of sample and 0.025 M $CaCl_2$ solution to determine APTT.

S1.7 *In vivo* hemostasis of cryogels

S1.7.1 A model of cylindrical (8 mm) defects in the liver of SD rats

The hemostatic function of CF/PD cryogels was examined by means of a generalized circular rat liver wound. The sterilized cryogels was prepared to a height of 10 mm and a diameter of 8 mm and compressed in a 1 mL sterile syringe for backup. SD rats were fed for 1 week, anesthetized with chloral hydrate, dehairing was performed on the abdomen near the thoracic cavity and slit with a scalpel, and the thoracic cavity of the rats was squeezed to expose the liver, which was gently wiped off with gauze to remove mucus on the surface of the liver. Weighed filter paper was padded under the liver, followed by rapid perforation of the rat liver with an 8 mm perforator to create a cylindrical noncompressible hemostatic wound. The hemostatic cryogels were rapidly injected into the defect, the hemostatic time was recorded, and the filter paper was weighed to calculate blood loss. SD rats without any treatment after surgery were used as blank controls. Medical gauze and commercially available gelatin sponges were used as controls.

S1.7.2 Power artery puncture model in SD rats

The hemostatic function of CF/PD cryogels were verified by needling the carotid artery of rats. The sterilized crystalline gel was prepared to a height of 5 mm and a diameter of 8 mm and compressed in a 1 mL sterile syringe for backup. After feeding the rats for 2 weeks, the rats were anesthetized, the body hair at the neck was removed, and the abdomen was cut open with a scalpel to search for the left carotid artery of the exposed rats. After puncturing the carotid vessels of rats with a needle penetration, the prepared syringe was quickly pushed to cover the carotid artery with cryogels. Record the time of hemostasis.

S1.8 Whole back wound healing experiment in rats of cryogels

A rat dorsal 8 mm wound model was used for wound healing experiments. After anesthetizing the rats, an 8 mm skin sampler was used to rotate on the back of the rats to establish a circular trauma wound, and cryogel with a thickness of 1mm-2 mm was added to the wound site and secured using a medical tape film. Blank control group with no treatment except medical tape. Medical gauze and commercially available gelatin sponges were used as controls. Remove wound scabs every other day, photograph and document, and replace hemostatic material. Until the wound was closed, the shrinkage area of the wound was calculated using ImageJ software to calculate the shrinkage ratio of the wound area. Hematoxylin-eosin (H&E) staining and Masson staining were performed on tissues collected from day 7 to assess epidermal regeneration, inflammatory response, and collagen deposition.

S1.9 Degradability of cryogels

S1.9.1 *In vitro* degradation

Configure 5 mg/mL of lysozyme solution, control the weight of crystalline gel at about 30 mg, weigh and submerge it in 10 mL of lysozyme solution, and incubate it at 37 °C in a constant temperature incubator. Replace the lysozyme solution with a fresh one every other day. The degradation ratio of cryogels were calculated based on the weight ratio after removing the cryogels at 5th day, 9th day, 13th day, 17th day, 21th day, and 25th day for freeze drying.

S1.9.2 *In vivo* degradation

After anesthetizing the rats, a wound of about 10 mm was made on the back of the rats with a scalpel, and a Co60 sterilized cryogel (3 mm high, 8 mm in diameter) was implanted into the subcutaneous tissue of the rats. On 7th day, 14th day, 21th day, and 28th day after surgery, three rats were randomly euthanized in each group, and the implants were removed and weighed to calculate the degradation ratio of the implants.

S2 Supporting Results

S2.1 CF/GMA Determination of grafting ratio

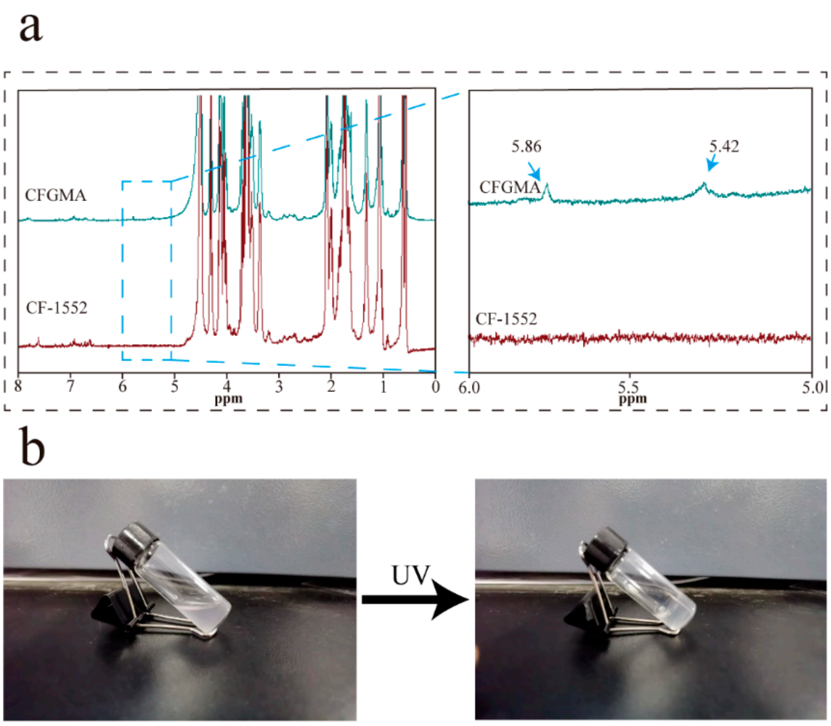

Figure S1. (a) 1H-NMR patterns of CFGMA and CF-1552. (b) UV curing study.

As shown in Figure S1a, the two new peaks at 5.42 and 5.86 chemical shifts are observed to be generated by two H vibrations on the C atom on the outside of the C=C double bond. At the same time a newly generated peak is observed on 1.24 for the H vibration on the hydroxyl group resulting from the ring opening of the epoxy group. The success of carbon-carbon double bond grafting was also confirmed by UV curing experiments (Figure S1b), where the solution was converted into hydrogel. In addition, the unsaturated amount of CF/GMA was measured to be 3.5 mmoL/g after quantitative iodine solution method, and the actual grafting ratio was 30.3% compared to the theoretical grafting ratio.

S2.2 Orthogonal experimental design

Table S1. Orthogonal experimental results.

Experimental	Factors			Shape recovery (%)	BCI (%)	Compressive strength (kPa)
	CFGMA (mg)	PEGDA (mL)	DMA (mg)			
1	150	0.2	50	85	40	42
2	150	0.4	0	50	55	65
3	150	0.4	100	75	36	64
4	150	0.6	50	70	48	73
5	175	0.2	0	65	50	87
6	175	0.2	100	70	31	78
7	175	0.4	50	100	25	120
8	175	0.6	0	50	56	110
9	175	0.6	100	80	35	98
10	200	0.2	50	70	45	84
11	200	0.4	0	45	52	120
12	200	0.4	50	60	50	141
13	200	0.4	100	70	40	101
14	175	0.4	50	100	25	121
15	175	0.4	50	100	24	120
16	175	0.4	50	100	24	121
17	175	0.4	50	100	24	121

Table S2 Analysis of the range and significance of orthogonal experimental results.

Factors	Norm	Mean 1	Mean 2	Mean 3	P-value	Distinctiveness
CFGMA	Shape restoration (%)	10.96	11.22	11.05	0.0097	**
PEGDA		9.83	9.92	9.88	0.0154	*
DMA		29.34	29.81	29.56	<0.0001	***
CFGMA	BCI (%)	3.46	3.71	3.54	0.0809	Ns
PEGDA		7.42	7.32	7.50	0.0036	*
DMA		25.10	25.21	25.45	<0.0001	***
CFGMA	Mechanical strength (kPa)	71.41	71.89	71.02	<0.0001	***
PEGDA		46.31	47.22	46.89	<0.0001	***
DMA		14.50	14.57	14.88	0.0623	Ns

The content of DMA had a significant effect on the strain recovery ratio, followed by the content of CF/GMA.

Adj R-Squared=0.9730, Pred R-Squared=0.8113, the difference between the two is less than 0.2, indicating successful modeling.

Among them, the content of CF/GMA and PEGDA had a significant shadow on the strain recovery ratio.

Adj R-Squared=0.9422, Pred R-Squared=0.7815, the difference between the two is less than 0.2, indicating successful modeling.

The content of DMA had the greatest effect on the coagulation index, followed by the content of PEGDA.

Adj R-Squared=0.9782, Pred R-Squared=0.8655, the difference between the two is less than 0.2, indicating successful modeling.

S2.3 FT-IR of CF-1552, CFGMA, and CF/PD

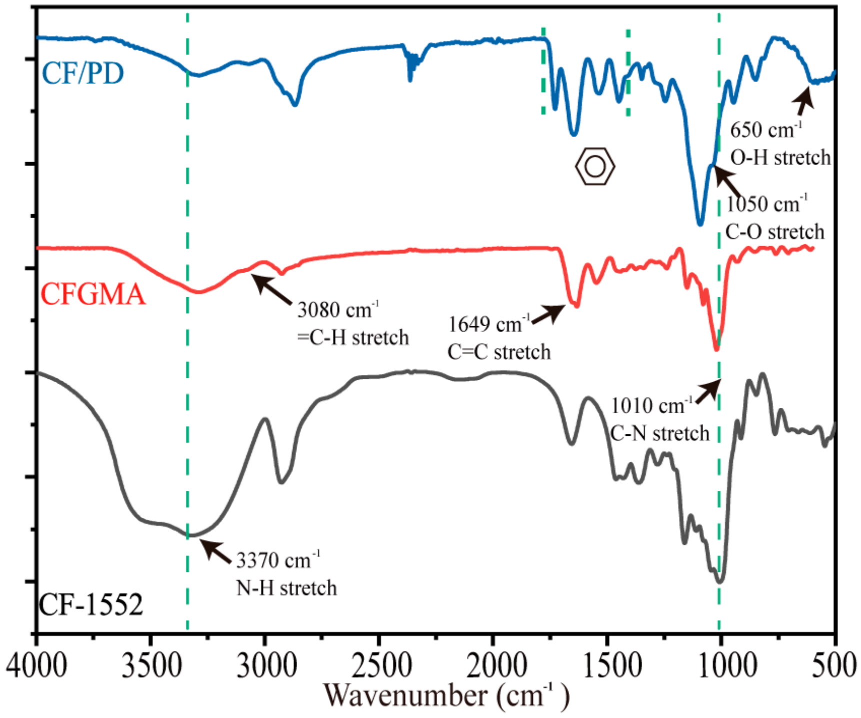

Figure S2. FT-IR mapping of CF-1552, CFGMA and CF/PD**.**

S2.4 Antimicrobial

The antimicrobial properties of cryogels were verified by in vitro antimicrobial experiments. In Figure S2, there was no decrease in the number of bacterial strains in the first two group (commercially available gelatin, cryogel without catechols) compared to the blank control, suggesting that they had no antibacterial properties. The last group showed a significant decrease in the number of bacterial strains, both Staphylococcus aureus (G+) and Escherichia coli (G-), with the addition of catechol moieties. It was demonstrated that cryogels incorporating catechol moieties have antimicrobial properties. The above results indicate that collagen covalently polymerized with polyethylene glycol sponges do not have antimicrobial properties, which are mainly provided by methacryloyl dopamine. The above results suggest that CF/PD cryogels has good potential for deep wound antimicrobial activity. Wounds are susceptible to bacterial infections when exposed to air, so cryogels adhesives need to provide not only hemostatic properties but also antimicrobial properties after hemostasis. In particular, deep wounds cannot be heated with infrared light to enhance antimicrobial properties and require the material to be inherently antimicrobial.

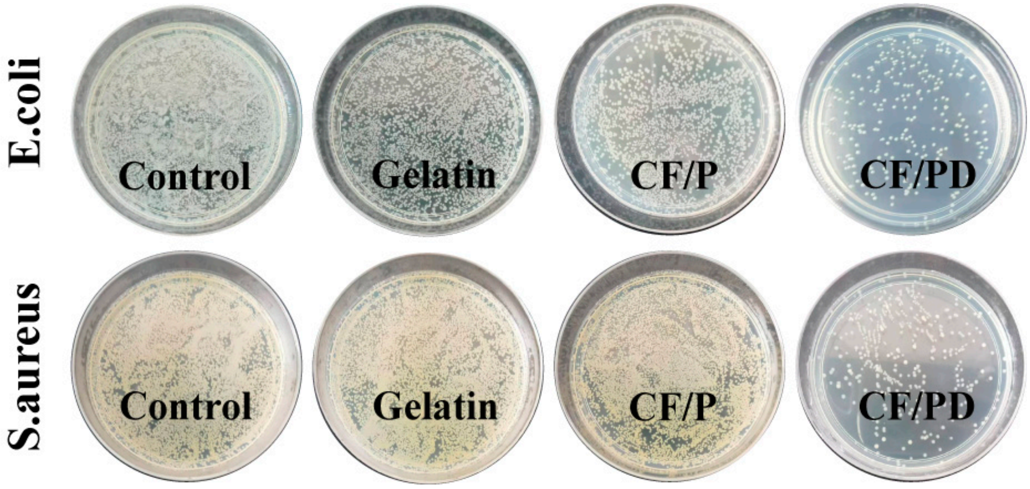

Figuer S3. Results of antimicrobial experiments by groups (CF/P is the experimental group without DMA).

S2.5 Water Absorption Recovery Video

PBS absorption.mp4

Video S1. PBS absorption.

Blood absorption.mp4

Video S2. Blood absorption.

S2.6 Video on Hemostasis

Rat liver hemostasis (1min)

Video S3. Rat liver hemostasis (1min).
